# Motives for choosing growth-enhancing hormone treatment in adolescents with idiopathic short stature: a questionnaire and structured interview study

**DOI:** 10.1186/1471-2431-5-15

**Published:** 2005-06-08

**Authors:** Hanneke Visser – van Balen, Rinie Geenen, Gerdine A Kamp, Jaap Huisman, Jan M Wit, Gerben Sinnema

**Affiliations:** 1 Department of Pediatric Psychology, University Medical Center Utrecht, Wilhelmina Children's Hospital, P.O. Box 85090, 3508 AB Utrecht, The Netherlands; 2 Department of Developmental Psychology, Utrecht University, P.O. Box 80140, 3508 TC Utrecht, The Netherlands; 3 Department of Health Psychology, Utrecht University, P.O. Box 80140, 3508 TC Utrecht, The Netherlands; 4 Department of Pediatrics, Gooi-Noord Hospital, P.O. Box 900, 1250 CA Laren, The Netherlands; 5 Department of Medical Psychology, VU University Medical Center, P.O. Box 7057, 1007 MB Amsterdam, The Netherlands; 6 Department of Pediatrics, Leiden University Medical Center, P.O. Box 9600, 2300 RC Leiden, The Netherlands

## Abstract

**Background:**

Growth-enhancing hormone treatment is considered a possible intervention in short but otherwise healthy adolescents. Although height gain is an obvious measure for evaluating hormone treatment, this may not be the ultimate goal for the person, but rather a means to reach other goals such as the amelioration of current height-related psychosocial problems or the enhancement of future prospects in life and society. The aim of our study was to clarify the motives of adolescents and their parents when choosing to participate in a growth-enhancing trial combining growth hormone and puberty-delaying hormone treatment.

**Methods:**

Participants were early pubertal adolescents (25 girls, 13 boys) aged from 11 to 13 years (mean age 11.5 years) with a height standard deviation score (SDS) ranging from -1.03 to -3.43. All had been classified as idiopathic short stature or persistent short stature born small for the gestational age (intrauterine growth retardation) on the basis of a height SDS below -2, or had a height SDS between -1 and -2 and a predicted adult height SDS below -2. The adolescents and their parents completed questionnaires and a structured interview on the presence of height-related stressors, parental worries about their child's behavior and future prospects, problems in psychosocial functioning, and treatment expectations. Questionnaire scores were compared to norms of the general Dutch population.

**Results:**

The adolescents reported normal psychosocial functioning and highly positive expectations of the treatment in terms of height gain, whereas the parents reported that their children encountered some behavioral problems (being anxious/depressed, and social and attention problems) and height-related stressors (being teased and juvenilized). About 40% of the parents were worried about their children's future prospects for finding a spouse or job. The motives of the adolescents and their parents exhibited rather different profiles. The most prevalent parental worries related to the current or future functioning of their children, while a few cases were characterized by no observed motives or by psychosocial problems only reported by the adolescents themselves.

**Conclusion:**

The motives for participating in a growth-enhancing hormone trial are more obvious in the parents than in the adolescents themselves. Two out of three parents report worries about the future opportunities or observe modest current psychosocial problems in their children. The adolescents want to gain height, but the motivation underlying this remains unclear. Few of the adolescents experience psychosocial problems. Our analyses revealed differences among individuals in terms of motives, which implies that in an evaluation of hormone treatment, the importance of divergent outcome variables will also differ among individuals. Effectiveness evaluations of hormone treatment to increase height and the consequential fulfillment of other goals must be awaited.

## Background

Growth-enhancing hormone treatment is considered a possible intervention in short but otherwise healthy adolescents. Although height gain is an obvious measure for evaluating hormone treatment, this may not be the ultimate goal of the person, but rather a means to reach other goals such as to ameliorate current height-related psychosocial problems or to enhance future prospects in life and society.

Psychosocial functioning has been emphasized in considerations of the motives of children with idiopathic short stature (ISS) and their parents seeking hormone treatment to achieve a height gain. Children who have never been medically referred do not seem to suffer from their stature [1-4]. Most studies involving medically referred children have revealed reduced social competence [5-14], while internalizing and externalizing behavior, body image, self-esteem, and scholastic competence have been shown, on average, to be between normal and below normal [5-8,10-17]. In addition to the possibility of improving psychosocial functioning, several other factors are hypothesized to motivate children and their parents to choose hormone treatment, including the experience of height-related psychosocial stressors (such as being teased or juvenilized), future expectations for finding a spouse and a desired career commensurate with intellectual potential and interests, and the expectation of a treatment-induced height gain.

Insight into the divergent motives at the time of choosing hormone treatment is important, because it will help in the choice of proper outcome measures for the evaluation of a trial. Insight into motivational factors is especially important in treatments combining biosynthetic growth hormone (GH) with a puberty-delaying hormone, because the possible benefits of enhancing growth must be balanced against the possible negative psychosocial consequences of delaying pubertal development. Moreover, such insight will clarify whether other treatments, such as psychosocial counseling, should be employed to solve current psychosocial problems and reach future goals.

Our study examined 38 medically referred early pubertal, short adolescents who agreed to participate in an experimental trial of a combined treatment of GH and a puberty-delaying hormone, and compared the results with short adolescents who received no intervention. Participants had a height standard deviation score (SDS) below -2, or between -1 and -2 with a predicted adult height SDS below -2, all without apparent underlying pathology. Twenty-six of the subjects had a normal birth weight and length and were classified as ISS [18], while 12 were born small for gestational age (SGA) [19,20]. Both ISS and SGA have been associated with problems in behavior, social functioning, school competence, and attention [21-23], while SGA has also been associated with lowered intelligence and minor neurologic dysfunctions [21,22,24].

With the aim of clarifying the motives of parents and children for participation in the trial, we examined the baseline scores of the adolescents on the following psychosocial factors before initiating the treatment: presence of height-related stressors, parental worries about the child's behavior and future prospects, self-reported problems in psychosocial functioning, and treatment expectations.

## Methods

### Study population and procedures

The inclusion criteria for participation in the study were as follows: being in early puberty as documented by Tanner breast stage 2 or 3 for girls and Tanner genital stage 2 or 3 for boys; an actual height SDS for the same age and sex of less than -2.0, or a height SDS between -1.0 and -2.0 with a predicted adult height of more than -2.0 SDS; a chronological age and bone age of less than 12 years for girls and 13 years for boys; a documented GH response of >20 mU/L (>10 ug/L) after a standard provocation test and/or during a sleep test; a ratio of sitting height/subischial leg height between the 3rd and 97th percentiles; and normal blood tests and urinalysis.

The study population consisted of 38 early pubertal adolescents (25 girls,13 boys) aged from 11 to 13 years (mean age 11.5 years). Most of the cohort (17 girls, 9 boys) had a normal birth size and were classified as ISS. Twelve adolescents (8 girls, 4 boys) were known to have had a birth length of more than 2 SD below the mean for gestational age and were classified as short-stature-born SGA. The most likely reason for the girls substantially outnumbering the boys is that the combination of short stature and early puberty is more common in girls than boys. The mean height of the adolescents was 134.9 (5.8) cm, with a range of 120.0–148.5 cm, corresponding to a height SDS ranging from -1.03 to -3.43. There were nine adolescents with an actual height SDS between -1.0 and -2.0 and a predicted adult height SDS of less than -2.0.

The following four Dutch hospitals participated in this multi-center study: the University Medical Center Utrecht-Wilhelmina Children's Hospital, the VU University Medical Center Amsterdam, the Sophia Children's Hospital Rotterdam, and the Catharina Hospital Eindhoven [19]. The first 40 referred adolescents who fulfilled the inclusion criteria and agreed to participate after having been informed about the study participated in our randomized controlled trial with puberty-delaying gonadotropin-releasing hormone agonist (GnRHa) therapy in combination with GH therapy or no therapeutic intervention. Twenty of the adolescents were randomly selected to receive the combination therapy for three years, while the other 20 adolescents were to receive no treatment. Immediately after randomization, two participants (one from the treatment group and one from the control group) decided to stop participation.

The experimental trial examined the hypothesis that the combination of GH and GnRHa for three years would increase the final height in comparison with untreated controls [19]. For three years the adolescents in the treatment group were given GH daily by subcutaneous injection of 4 IU (1.33 mg) per square meter body surface; (Genotropin, Pharmacia, Sweden; now Pfizer, New York, USA) and a depot preparation of 3.75 mg of GnRHa (Decapeptyl-CR, Ferring, Sweden) every four weeks intramuscularly. The adolescents in the control group were followed yearly to document their growth and pubertal development [19]. Before treatment, and at one, two, and three years after beginning the treatment, the adolescents and the parents of both the treatment and control groups filled out questionnaires to assess the psychosocial functioning of the adolescents. The parents were also interviewed.

A skilled psychologist examined the adolescents and their parents in the interval between randomization and starting treatment. This interval was chosen for ethical reasons to avoid the suggestion that the answers of the parents or adolescents might affect the group allocation. Moreover, providing knowledge of the result of the randomization procedure to both parents and adolescents was assumed to make them better able to give unbiased responses to the questionnaires and interview. The adolescents filled out questionnaires on perceived competence, psychological distress, body image, self-image, and personality characteristics. The parents filled out questionnaires regarding emotional and behavioral problems of their children, and were interviewed by a psychologist.

The medical ethics committees at the four participating centers approved the study. The parents of all children provided written informed consent before participation.

### Measures

#### Parental reports

A structured interview was conducted with parents to assess the adolescents' health-related development, current height-related stressors, and parental concerns about their child's future. Parents were asked about health problems, the child's age at which a physician was consulted for the first time because of the growth retardation, and who initiated the referral (school doctor, family doctor, or pediatrician). In order to determine the height-related psychosocial stressors encountered by the adolescents, the parents were asked whether their children were teased or juvenilized by other children. To find out parental worries about the future of their child, parents were asked whether they considered that the prospects of their child were equal to that of persons of normal height in the labor market and finding a spouse (yes, doubtful, no).

To assess behavioral and emotional problems, the parents filled out the Child Behavior Check List (CBCL) [25]. This questionnaire consists of the following eight syndrome areas (the so-called narrow-band scales): withdrawn behavior, somatic complaints, anxious/depressed behavior, social problems, thought problems, attention problems, delinquent behavior, and aggressive behavior. The scores of the subscales were summarized into a score for internalizing and externalizing problems, and a total problem score (the so-called broad-band scales).

The Silhouette Apperception Technique (SAT) has been shown to be a valid and useful instrument for assessing perceptions of the present and future heights of adolescents [7,26-28]. The parents were shown drawings of people of different heights, corresponding proportionally with the heights of the 3rd, 25th, 50th, 75th, and 97th percentiles, and asked to identify the drawings that they thought best fit the current and future heights of their child.

#### Adolescents' self-reports

The adolescents completed a global intelligence test and a series of questionnaires regarding perceived competence, psychological distress, and personality characteristics.

The Dutch versions of the Self-Perception Profile for Children (CBSK) and the Self-Perception Profile for Adolescents (CBSA) were used [29,30]. The CBSK consists of six perceived competence subscales: scholastic competence, social acceptance, athletic competence, physical appearance, behavior and conscience, and global self-worth. The CBSA consists of these six scales plus the scales of friendship and romantic love. In our study we only used the six scales that the CBSK and the CBSA have in common.

Anxiety was measured using the Dutch version of the State-Trait Anxiety Inventory for Children (ZBV-K), with the subscales of state anxiety and trait anxiety [31]. Depressive mood was assessed by the KDVK (Dutch Short Depression Questionnaire for Children) [32].

Personality characteristics of the adolescents were investigated using the Dutch Personality Questionnaire-Junior (NPV-J), with five subscales: inadequacy, perseverance, social inadequacy, recalcitrance, and dominance [33].

The SAT was used to measure perceptions of the present and future heights of the adolescents [7,26-28].

Intelligence was assessed by the Dutch short version of the Wechsler Intelligence Scale for Children (Revised) (WISC-Rn) [34].

### Data analyses

Standard deviation scores (SDS) were used to compare the results of the quantitative questionnaires with the means of the general Dutch population. This was performed by subtracting the mean score of the norm group of the same age and sex from the score of the participants, with this difference divided by the SD of the norm group. This score expresses how much each study subject deviates from the norm in SD units. *T*-tests examined whether these age-and sex-adjusted scores significantly deviated from zero (i.e., from the norm).

To categorize groups of adolescents with divergent motives for wanting hormone treatment, we formed subgroups based on the presence of height-related stressors (being teased or juvenilized), parental worries about future prospects (regarding finding a spouse or job), parental worries about their child's behavior (internalizing or externalizing problems), and self-reported problems in psychosocial functioning (low self-esteem, anxiety, or depressive mood). The cut-off score for having externalizing or internalizing problems was a norm deviation score of 1 SD higher than the mean for the same age and sex on the CBCL scales for internalizing and externalizing behavioral problems. This score corresponds with a CBCL T-score of 60 (84th percentile) in the healthy norm group, and conforms with CBCL standards for the borderline range of clinical problems [25]. The cut-off score for anxiety and low self-esteem was a norm deviation score of at least 1 SD higher (anxiety) or lower (self-esteem) than the means for the same age and sex on the trait anxiety and the global self-worth scale, respectively. The cut-off score for indicating clinical signs of depression was a raw score of 4 points or more on the depression scale [32].

Our study group was small and significant differences were generally not evident in the results between boys and girls, between adolescents with ISS and those born SGA, and between adolescents who were to receive hormone treatment and those who were not. Hence our report focuses primarily on the results for the whole group.

## Results

### Medical referral

The mean age of the child when a physician was consulted for the first time because of the growth retardation was 7.3 years (SD = 3.5 years). Medical referral was initiated by the family physician (53%; seven adolescents with SGA, thirteen with ISS) or school physician (23.5%; two adolescents with SGA, seven with ISS), whereas some of the adolescents were already under supervision of a pediatrician as young children because of health problems other than short stature (23.5%; three adolescents with SGA, six with ISS). Ninety percent of the parents reported that their children had never experienced serious health problems.

### Height-related psychosocial stressors and future expectations

Approximately one-quarter (28%) of parents reported that their child was teased by peers because of short stature (15% of boys, 35% of girls), and 14% of parents reported that their child was juvenilized by peers (23% of boys, 9% of girls). All but one adolescent took part in gymnastic lessons at school (97%).

Regarding the effects of short stature on future expectations, 44.5% of the parents expected their child to have a lower prospect in the labor market as an adult (39% of boys, 48% of girls), and 39% expected their child to have a lower prospect of finding a spouse (77% of boys, 17% of girls). This difference between boys and girls was significant (*p *< 0.01). No other significant differences between boys and girls were found.

### Psychosocial functioning

#### Parental reports

On parental ratings of behavioral difficulties as measured by the CBCL, the short adolescents exhibited higher scores than the general Dutch norm group on the broad-band scales of internalizing problems (*p *< 0.05) and total behavioral problems (*p *< 0.05) (Table 1). The elevated score for withdrawn behavior was marginally significant (*p *< 0.10), while the higher score for anxious/depressed behavior was significant (*p *< 0.05). These narrow-band scales are summarized in the internalizing scale. The short adolescents also exhibited higher than normal scores on the social problems (*p *= 0.01) and attention problems (*p *< 0.05) scales. The scores deviated by 0.50–0.73 SDS from the norm (see Table 1), which reflects moderate effects. Three adolescents were in the clinical range for internalizing problems (CBCL T-score of 63; 90th percentile). The short adolescents did not score significantly higher than the norm group on the narrow-band scales of somatic complaints, thought problems, delinquent behavior and aggressive behavior, or the broad-band scale of externalizing problems. One adolescent scored in the clinical range for externalizing behavior.

**Table 1:** Emotional and behavioral problems of 34 adolescents with short stature as judged by their parents.

Variable	*d*	SD	*t*	*p*	
Withdrawn behavior	0.34	1.10	1.82	0.08	
Somatic complaints	0.38	1.34	1.66	0.11	
Anxious/depressed behavior	0.54	1.35	2.35	0.03	*
Social problems	0.73	1.44	2.96	0.01	**
Thought problems	0.26	1.00	1.49	0.15	
Attention problems	0.59	1.36	2.54	0.02	*
Delinquent behavior	0.29	1.46	1.18	0.25	
Aggressive behavior	0.26	1.03	1.47	0.15	
Internalizing problems	0.54	1.31	2.42	0.02	*
Externalizing problems	0.28	1.17	1.40	0.17	
Total behavioral problems	0.50	1.25	2.35	0.03	*

Mean scores (*d*), standard deviations (SD), and *t *and *p *values. The *d *values reflect the deviations from the Dutch normative population in standard deviation units, where a positive score indicates that the adolescents with short stature are judged to have more problems than the norm group.

The *d *values have the following common effect sizes: a value smaller than 0.2 reflects no deviation from the norm, while values between 0.2 and 0.5, between 0.5 and 0.8, and greater than 0.8 reflect small, moderate, and large deviations, respectively [42]. *T*-tests examined whether norm deviation scores deviated from zero (the norm).

* *p *< 0.05, ** *p *< 0.01

To clarify the nature of the problems perceived by the parents, we examined specific items on the CBCL scales where parents observed significant problems. Items for which the adolescents deviated at least moderately from the norm included generic items on the scale of anxious/depressed behavior, height-related items on the scale of social problems, and both generic and height-related items on the scale of attention problems (for the specific items, see Table 2).

**Table 2:** Scores at separate items of the CBCL scales anxious/depressed behavior, social problems, and attention problems.

Item	*d*	SD	*t*	*p*	
Anxious/depressed behavior:					
112. Worries	0.83	1.36	3.55	0.001	**
45. Nervous, highstrung, or tense	0.65	1.25	3.03	0.005	**
35. Feels worthless or inferior	0.63	1.66	2.22	0.03	*
50. Too fearful or anxious	0.42	1.53	1.59	0.12	
12. Complaints of loneliness	0.37	1.49	1.44	0.16	
32. Feels he/she has to be perfect	0.35	1.03	1.98	0.06	
103. Unhappy, sad, or depressed	0.33	1.21	1.57	0.13	
14. Cries a lot	0.19	1.30	0.86	0.40	
52. Feels too guilty	0.14	1.47	0.54	0.59	
33. Feels or complaints that no one loves him/her	0.11	1.08	0.60	0.55	
71. Self-conscious or easily embarrassed	0.11	1.19	0.53	0.60	
89. Suspicious	0.08	0.98	0.45	0.65	
31. Fears he/she might think or do something bad	-0.03	0.89	-0.21	0.84	
34. Feels others are out to get him/her	-0.11	0.80	-0.83	0.41	
Social problems:					
64. Prefers being with younger kids	1.06	1.67	3.71	0.001	**
38. Gets teased a lot	0.91	1.38	3.86	0.000	**
1. Acts too young for his/her age	0.65	1.39	2.73	0.010	**
11. Clings to adults or too dependent	0.30	1.31	1.33	0.19	
55. Overweight	0.03	1.00	0.20	0.85	
48. Not liked by other kids	-0.08	0.81	-0.58	0.57	
62. Poorly coordinated or clumsy	-0.10	0.75	-0.75	0.46	
25. Doesn't get along with other kids	-0.15	0.57	-1.56	0.13	
Attention problems					
1. Acts too young for his/her age	0.65	1.39	2.73	0.010	**
45. Nervous, highstrung, or tense	0.65	1.25	3.03	0.005	**
8. Can't concentrate, can't pay attention for long	0.42	1.20	2.07	0.047	*
46. Nervous movements or twitching	0.39	1.55	1.48	0.15	
41. Impulsive or acts without thinking	0.31	1.23	1.48	0.15	
13. Confused or seems to be in a fog	0.24	1.27	1.12	0.27	
61. Poor school work	0.17	1.23	0.82	0.42	
10. Can't sit still, restless, or hyperactive	0.15	0.99	0.90	0.37	
17. Day-dreams or gets lost in his/her thoughts	0.14	1.05	0.77	0.45	
80. Stares blankly	0.06	1.11	0.31	0.76	
62. Poorly coordinated or clumsy	-0.10	0.75	-0.75	0.46	

Mean scores (*d*), standard deviations (SD), and *t *and *p *values. The *d *values reflect the deviations from the Dutch normative population in standard deviation units, where a positive score indicates that the adolescents with short stature are judged to have more problems than the norm group.

The *d *values have the following common effect sizes: a value smaller than 0.2 reflects no deviation from the norm, while values between 0.2 and 0.5, between 0.5 and 0.8, and greater than 0.8 reflect small, moderate, and large deviations, respectively [42]. *T*-tests examined whether norm deviation scores deviated from zero (the norm). * *p *< 0.05, ** *p *< 0.01

Note that some items of the CBCL load on more than one scale

#### Adolescents' self-reports

Virtually none of the scores of the short adolescents on self-reported questionnaires differed significantly from the norm group (Table 3). Adolescents with ISS or SGA did not deviate from the norm group with respect to perceived competence and psychological distress. Indeed, their perceived social acceptance was higher than that in the norm group (*p *= 0.05). With respect to personality characteristics, adolescents with ISS or SGA described themselves to be more persistent (quiet, conscientious, and diligent) than the norm group (*p *< 0.05). Two adolescents were in the clinical range (deviation of more than 2SDs) for global self-worth. One adolescent was at the clinical level for trait anxiety.

**Table 3:** Psychological functioning and distress as reported by the adolescents with short stature.

Variable	*n*	*d*	SD	*t*	*p*	
Perceived competence (CBSK):						
Scholastic competence	31	0.15	1.06	0.78	0.45	
Social acceptance	31	0.37	1.00	2.04	0.05	*
Athletic competence	31	0.22	1.11	1.08	0.29	
Physical appearance	31	-0.21	0.98	-1.22	0.23	
Behavior/conscience	31	0.17	1.05	0.90	0.38	
Global self-worth	31	0.02	1.08	0.07	0.94	
Psychological distress (ZBV-K):						
State anxiety	37	-0.01	1.06	-0.08	0.94	
Trait anxiety	37	0.09	1.12	-0.52	0.61	
Personality characteristics (NPV-J):						
Inadequacy	38	-0.08	0.88	-0.52	0.61	
Perseverance	38	0.34	0.95	2.20	0.03	*
Social inadequacy	38	0.05	0.82	0.36	0.72	
Recalcitrance	38	-0.05	0.97	-0.32	0.75	
Domination	38	0.23	1.07	1.34	0.19	

Mean scores (*d*), standard deviations (SD), and *t *and *p *values. The *d *values reflect the deviations from the Dutch normative population in standard deviation units, where a positive score indicates that the adolescents with short stature judged themselves to have higher perceived competence, more anxiety, and a higher score on personality scales than the norm group, respectively.

The *d *values have the following common effect sizes: a value smaller than 0.2 reflects no deviation from the norm, while values between 0.2 and 0.5, between 0.5 and 0.8, and greater than 0.8 reflect small, moderate, and large deviations, respectively [42]. *T*-tests examined whether norm deviation scores deviated from zero (the norm).

* *p *< 0.05, ** *p *< 0.01

### Cognitive functioning

Intelligence scores (corrected total WISC-Rn scores) ranged from 66 to 128, with a mean score of 99.8. Six adolescents (three ISS, three SGA) had an intelligence lower than 80. The intelligence did not differ significantly between adolescents with ISS and those with SGA.

### Expectations of hormone treatment: perception of current and future heights

Perceptions of current and future heights were analyzed separately in adolescents who were to receive hormone treatment and those who were not (Table 4). Most parents and adolescents in both the treatment and control groups estimated the current height of the adolescent at the 3rd percentile of the population references, which corresponds well to their actual height. Parents of the adolescents in the treatment group expected a greater future height than the parents of the adolescents in the control group (*Z *= -2.68, *p *= 0.007). None of the other comparisons between the experimental and control groups revealed significant differences.

**Table 4:** Current and future heights as perceived by adolescents and their parents on the Silhouette Apperception Technique

Treatment group (*n *= 19)	Current height (%)		Future height (%)	
Percentile	Adolescents	Parents	Adolescents	Parents

3^rd^	52.6	88.9	5.3	0.0
25^th^	36.8	5.6	15.8	38.9
50^th^	5.3	5.6	26.3	38.9
75^th^	0.0	0.0	42.1	11.1
97^th^	5.3	0.0	10.5	11.1

Control group (*n *= 19)	Current height (%)		Future height (%)	

Percentile	Adolescents	Parents	Adolescents	Parents

3^rd^	47.4	77.8	15.8	50.0
25^th^	47.4	16.7	26.3	22.2
50^th^	5.3	5.6	15.8	11.1
75^th^	0.0	0.0	42.1	16.7
97^th^	0.0	0.0	0.0	0.0

### Groups classified according to motives

To provide a summary description, four groups of adolescents were distinguished based on the presence of height-related stressors, parental worries about future prospects, parental worries about their children's behavior, and self-reported problems in psychosocial functioning (Table 5).

**Table 5:** Classification of adolescents based on motives

	Parental reports			Self-reports
	Height-related stressors	Worries about future prospects	Behavioral problems	Psychosocial problems

Group 1 (*n *= 4)	-	-	-	-

Group 2a (*n *= 3)	-	+	-	-
Group 2b (*n *= 4)	+	-	-	-
Group 2c (*n *= 4)	+	+	-	-

Group 3a (*n *= 4)	-	+	+	-
Group 3b (*n *= 7)	+	+	+	-

Group 4a (*n *= 6)	-	-	-	+
Group 4b (*n *= 2)	-	-	+	+

The presence or absence of a motive is indicated by '+' and '-', respectively; motives include parental reports of the presence of height-related stressors (being teased or juvenilized), worries about future prospects (finding a spouse or job), and behavioral problems (internalizing or externalizing problems), and self-reporting of psychosocial problems (anxiety, low self-worth, or depressive mood). The four missing cases are due to one of the classifying variables being missing.

Group 1 consisted of four adolescents and their parents (12%) who did not report any psychosocial problems. However, all adolescents in this group reported having high expectations of the treatment in terms of height gain (these data are not listed in Table 5).

Group 2 consisted of 11 adolescents (32%) whose parents reported height-related psychosocial stressors or worries about future prospects, but no problems in psychosocial functioning (parental or adolescents' reports).

Group 3 consisted of 11 adolescents (32%) whose parents reported problems in psychosocial functioning as well as worries about future prospects and in most cases the presence of height-related psychosocial stressors. The adolescents themselves did not report problems in psychosocial functioning.

Group 4 consisted of eight adolescents (24%) who reported problems in psychosocial functioning, while their parents did not report height-related psychosocial stressors and worries about future prospects, and in most cases did not report behavioral problems.

## Discussion

In a medically referred group of early pubertal adolescents with ISS or SGA, the motivation of the adolescents and their parents for choosing hormone treatment was investigated before initiating a combined GH and puberty delaying hormone treatment.

Parental reports revealed that current height-related psychosocial stressors were not the main reason for wanting growth-enhancing hormone treatment. Some of the parents reported that their children were teased (28%) or juvenilized (14%) because of their stature. These findings are close to those from another Dutch study [7], but in contrast with an American study that showed teasing and juvenilizing rates of 50% and 70%, respectively [14]. According to their parents, most of the adolescents in the present study were relatively free of current stressors. More than 40% of the parents expected that their children would have a decreased prospect in the labor market or difficulties in finding a spouse. This suggests that the motivation of providing opportunities for the future of the adolescents was a compelling reason for parents to choose hormone treatment.

Another possible reason for wanting hormone treatment is parental worries about the psychosocial functioning of their children. On average, as judged by their parents, the adolescents encountered internalizing symptoms, such as anxious or depressed tendencies, as well as social and attention problems. These problems were of a moderate magnitude compared to Dutch norms. It is likely that our analysis overestimated the actual psychosocial dysfunctioning, because the normative criteria were based on a very healthy group: any child who had been referred to a mental-health professional in the past 12 months, or who was currently receiving special educational services, was excluded from the normative sample [25,35]. Moreover, some of these problems are height-related issues that need not be a behavioral problem, such as preferring to be with younger kids or acting too young for his/her age.

The perceptions of adolescents about their own psychosocial functioning did not confirm the parental worries. The adolescents reported normal competence and personality, and even higher competence on social acceptance and perseverance, and little distress. This raises the question of whether adolescents or their parents are the best judges about well-being and functioning of adolescents. Adolescents may be unreliable informants because they are too young to give an adequate assessment of their own functioning, lack a time perspective, or have a tendency toward denial, while parents may be unreliable because of unrealistic anxieties about the health, future, and behavior of their children [36,37]. Our observation of more psychosocial problems being reported by parents than adolescents suggests that the perception of psychosocial problems is a stronger motive for parents than for adolescents when choosing to participate in the hormone treatment trial.

The perception of the current height of the adolescents was accurate in the majority of parents, while several adolescents had a tendency to overestimate their current height. Consistent with previous observations [26,38], several adolescents who were to receive hormone treatment had unrealistic expectations of their future height (as did their parents). Even those who were not to receive hormone treatment had high expectations of their future height. Optimism, even when unrealistic, has been shown to motivate the choice for a treatment and its adherence once started in several diseases [39,40]. However, unrealistic expectations may also be associated with a poor psychosocial outcome, as has been demonstrated in persons seeking cosmetic surgery, for example [41].

The tentative breakdown of subgroups provides a descriptive summary of four rather different profiles of motives for hormone treatment in the adolescents and their parents. A small group of adolescents and their parents reported no psychosocial problems. Highly positive treatment expectations of the adolescents in terms of height gain was the only detected motive, with the underlying reason remaining unclear. Perhaps it predominantly reflects the developmental wish of any child to want to grow (up). The parents reported height-related stressors or psychosocial problems and in most cases these worries about current problems were accompanied by worries about future prospects. The final group consisted of adolescents who reported problems in their psychosocial functioning, while their parents did not necessarily observe problems and were not worried. The cause of these problems and the relation with height remain unclear. In choosing such an intensive hormone treatment involving daily injections, pubertal delay, and possible side effects, we would have expected at least a subgroup of cases to show a motivation in parents as well as adolescents. However, none of the adolescents exhibited elevated scores on all motives, and only two pairs of parents and adolescents were congruent with respect to the observation of psychosocial problems. In the majority of cases it was either the parents or the adolescents who reported one or more motives.

Our sample size was sufficiently large to allow conclusions to be drawn regarding the comparison with normative data, but a larger sample size is needed for examining with sufficient power the possible roles of gender, type of short stature, and risks and protective factors that may modulate the psychosocial functioning of these adolescents [42].

## Conclusion

Our study demonstrates that the motives of parents to let their children participate in a growth-enhancing hormone trial are more obvious than the motives of the early pubertal adolescents themselves. Two out of three parents reported worries about the future opportunities or observed modest current psychosocial problems in their children. The adolescents wanted to gain height, but the underlying motivation remains unclear. Few of the adolescents experienced psychosocial problems. Our analyses showed that motives varied among individuals. This result implies that when evaluating hormone treatment, the importance of divergent outcome variables will also differ among individuals. Effectiveness evaluations of hormone treatment to increase height and the consequential fulfillment of other goals must be awaited.

## Abbreviations

ISS idiopathic short stature

SGA small for gestational age

SDS standard deviation score

GH growth hormone

GnRHa gonadotropin-releasing hormone agonists

CBCL Child Behavior Check List

SAT Silhouette Apperception Technique

CBSK/A Dutch version of the Self-Perception Profile for Children/Adolescents

ZBV-K Dutch version of the State-Trait Anxiety Inventory for Children

KDVK Dutch Short Depression Questionnaire for Children

NPV-J Dutch Personality Questionnaire-Junior

WISC-Rn Dutch short version of the Wechsler Intelligence Scale for Children (Revised)

## Competing interests

The author(s) declare that they have no competing interests.

## Authors' contributions

HVB and RG analyzed the data and wrote the manuscript together with GS (the project supervisor) who, along with JH, was also involved in the study design and acquisition of data. GAK and JMW (the medical supervisor) were responsible for the design and execution of the medical part of the study. All authors critically revised the manuscript, and read and approved the final version.

## Pre-publication history

The pre-publication history for this paper can be accessed here:


